# The Role of Exosomes in Thyroid Cancer and Their Potential Clinical Application

**DOI:** 10.3389/fonc.2020.596132

**Published:** 2020-12-01

**Authors:** Kaixiang Feng, Runsheng Ma, Lele Zhang, Hongqiang Li, Yifeng Tang, Gongbo Du, Dongpeng Niu, Detao Yin

**Affiliations:** ^1^ Department of Thyroid Surgery, The First Affiliated Hospital of Zhengzhou University, Zhengzhou, China; ^2^ Department of Thyroid Surgery, Key Discipline Laboratory of Clinical Medicine of Henan, Zhengzhou, China; ^3^ Academy of Medical Sciences, Zhengzhou University, Zhengzhou, China

**Keywords:** extracellular vesicles, exosome, thyroid cancer, biomarker, oncology treatment

## Abstract

The incidence of thyroid cancer (TC) is rapidly increasing worldwide. The diagnostic accuracy and dynamics of TC need to be improved, and traditional treatments are not effective enough for patients with poorly differentiated thyroid cancer. Exosomes are membrane vesicles secreted specifically by various cells and are involved in intercellular communication. Recent studies have shown that exosomes secreted by TC cells contribute to tumor progression, angiogenesis and metastasis. Exosomes in liquid biopsies can reflect the overall molecular information of tumors, and have natural advantages in diagnosing TC. Exosomes also play an important role in tumor therapy due to their special physicochemical properties. TC patients will benefit as more exosome patterns are discovered. In this review, we discuss the role of TC-derived exosomes in tumorigenesis and development, and describe the application of exosomes in the diagnosis and treatment of TC.

## Introduction

Thyroid cancer (TC), one of the fastest-growing cancers in the world, has become the most common malignancy of the endocrine system in recent years (1–3). More than 90% of TCs diagnosed annually are differentiated thyroid cancer (DTC), which histologically includes papillary thyroid cancer (PTC) and follicular thyroid cancer (FTC), whereas the remaining subset of TC comprises medullary thyroid cancer (MTC) and anaplastic thyroid cancer (ATC); however, these two subtypes are associated with a poor prognosis (4, 5). Through common treatment methods, including surgery, radioactive iodine therapy, chemotherapy, external irradiation, and targeted therapy, most cases of TC have high cure rates and low tumor recurrence (6). However, there are still not adequate treatment options for advanced TC (7, 8). The median survival time of patients with ATC is less than 6 months regardless of stage. Although aggressive therapy has been used, it has not significantly changed in recent years (9). The exploration of new treatment strategies is mandatory to improve the survival of these patients and guarantee good quality of life (8, 10).

Extracellular vesicles (EVs) are heterogeneous groups of vesicles with membranous subcellular structures released by almost all cell types. They can be found in various physiological fluids including blood, urine, saliva, lymph, and seminal fluid (11, 12). Based on the origin and diameter of vesicles, EVs can be divided into three major subclasses: exosomes, microvesicles, and apoptotic bodies. Exosomes are of endosomal origin and range in size from 30 to 120 nm and can be released by both eukaryotic and prokaryotic cells (13–15). Exosomes produced by tumor cells can create favorable conditions for tumor progression, such as induction of the angiogenic switch, increased vascular permeability, enhanced drug resistance and instigation of immune escape (16, 17). In addition, different types of cells, including mesenchymal stem cells (MSCs), adipocytes, fibroblasts, and immune cells in the tumor microenvironment (TME), can also induce the release of exosomes that promote tumor progression (18, 19). Understanding the role of exosomes in these cells will help unravel the mechanism underlying cancer. Furthermore, exosomes and their functions will provide more ways to diagnose, predict, and treat cancer (20). The utility of exosome detection in liquid biopsies is particularly promising for TC, and exploration of the use of exosomes as vehicles for the delivery of drug payloads is actively in progress. With the vast amount of information on exosomes emerging in the literature, these particles will likely be increasingly used in organisms. This review describes the function, separation methods, and biomedical applications of exosomes as well as current information on exosomes in TC.

## The Biogenesis and Isolation of Exosomes

The general understanding of EVs continued to improve for decades since they were first defined as "platelet dust" in the 1960s (21). There has been a paradigm shift from garbage carriers to new modes of intercellular communication and clinical applications (22–24). EVs are nanoparticles surrounded by lipid membranes that contain proteins, lipids, metabolites, and biologically active nucleic acids, which include DNA, mRNA, and noncoding RNA (ncRNA). These contents can be transported to recipient cells and interact with them to trigger biological responses (25, 26). In response to various internal and external conditions, the molecular properties and contents of EVs are often altered (11). Exosomes are an important class of EVs and an attractive tool for diagnosing and treating diseases, with special biological significance. Exosomes play critical roles in a variety of pathways, including cancer (27), cardiovascular disease (28), nervous system disorders (29), infectious diseases (30), and immunological diseases (31). However, normal bodily functions are also dependent on exosomes (32). For example, exosomes can protect host cells *in vitro* by acting as scavenging agents for a variety of toxins (33, 34). TSHR exosomes from patients with Graves' disease can reduce autoantibody-mediated activation of thyroid function (35). The dichotomous behavior and plasticity of exosomes have encouraged researchers to put more efforts into manipulating exosomes and determining how they can harm or benefit an organism.

**Figure 1:**
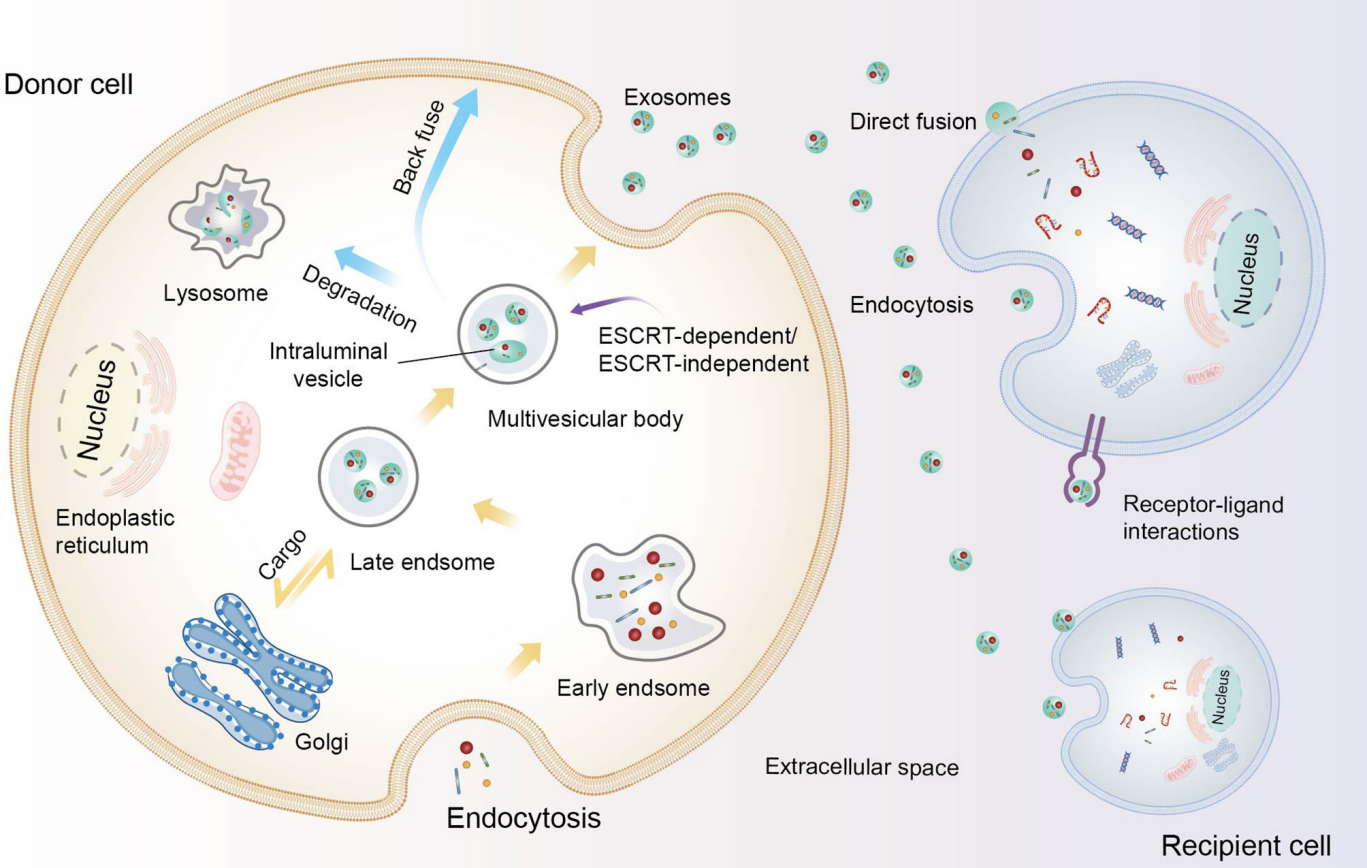
The biogenesis of exosomes and their communication with recipient cells. Constituents from the extracellular space enter the cell by endocytosis, leading to the formation of early endosomes and gradually maturing into late endosomes. After the endosomes undergo inward budding and the cargo is further packaged, intraluminal vesicles (ILVs) form and constitute multivesicular bodies (MVBs). Subsequently, MVBs degrade in lysosomes, are transported back to the plasma membrane, or fuse with the plasma membrane to release ILVs as exosomes. These loaded exosomes release proteins, nucleic acids, and other contents to recipient cells though direct fusion, internalization, and ligand-receptor interactions.

Exosomes arise from either intraluminal vesicles (ILVs) released after multivesicular bodies (MVBs) fusion with the plasma membrane or other means (22, 27, 32) (**Figure 1**). The formation of exosomes begins with the emergence of the initial endosomes through the endocytosis of extracellular components and then their gradual maturation into late sorting endosomes before they finally become MVBs, which contain several ILVs (22, 36). Concomitant with this process, proteins, DNA and RNA can be selectively loaded into MVBs, after which the exosomes are released into the extracellular space by MVBs. Furthermore, MVBs can also be degraded by fusion with lysosomes or recovered by the plasma membrane through the back-fusion pathway (32, 37). The released exosomes can deliver cargo to target cells through endocytosis, receptor-ligand interactions, and fusion with the plasma membrane. Indeed, the biogenesis of exosomes and the sorting of exosome cargoes are complex and worthy of further investigation. Both lipids, such as lysophospholipids and ceramides, and proteins, such as CD81, CD9, Rab, Ral, Alix, and the endosomal sorting complexes required for transport (ESCRT), participate in these processes (20, 27, 37). It should be emphasized that protein classification in endosomes is crucial for protein stability, endosome maturation, etc., and the ESCRT mechanism is an important contributor to this activity (38). ESCRT located on the MVB membrane encompasses four complexes: 0, I, II, and III. ESCRT-0 combined with ESCRT-I recruits ESCRT-II and cargoes, and then ESCRT-II recruits ESCRT-III to assist with ILV budding. Other studies have shown that proteins containing Bro1 domains, such as ALIX, HD-PTP, and yeast Bro1, can bypass ESCRT-II and ESCRT-I and promote the assembly of ESCRT-III components (38–40). Interestingly, there are also ESCRT-independent mechanisms that can influence exosome occurrence. For example, RAB31 drives the formation of ILV independently of the ESCRT machinery and tetraspanin pathway (41). In general, multiple molecules and mechanisms are involved in the biological processes of exosomes, but their control pathways are still poorly understood. Due to their cellular origin, intrinsic biology, microenvironment and other factors, the size, content, inclusion, and function of exosomes may also differ (20). Basic research on exosomes is key for their clinical development; however, technical barriers to exosome extraction procedures limit the basic and applied research of exosomes to a certain extent (42).

Current exosome separation strategies include ultracentrifugation, ultrafiltration, size-exclusion chromatography, immunoaffinity capture, charge neutralization-based polymer precipitation, and microfluidic techniques, but none of them are sufficient for high-yield extractions (43, 44). Although ultracentrifugation is the most widely used primary isolation method for exosomes, it is also susceptible to contamination, and has low reproducibility, and low sample throughput (45, 46). Exosome heterogeneity also remains a research challenge. Because the current exosome separation technology is still limited and there is no particularly uniform standard, the term "exosome" used in this paper is not specifically distinguished from a mixture of EVs, as suggested in the guidelines of the International Society for Extracellular Vesicles (ISEV) (47). Appropriate separation techniques or combination applications will help researchers solve many of the problems facing exosome research (43). Standardized exosome separation criteria will need to be developed in the future to meet the growing need for the specific screening of different sample types and for accurate exosome characterization.

## Exosome Biomarkers for TC

**Figure 2:**
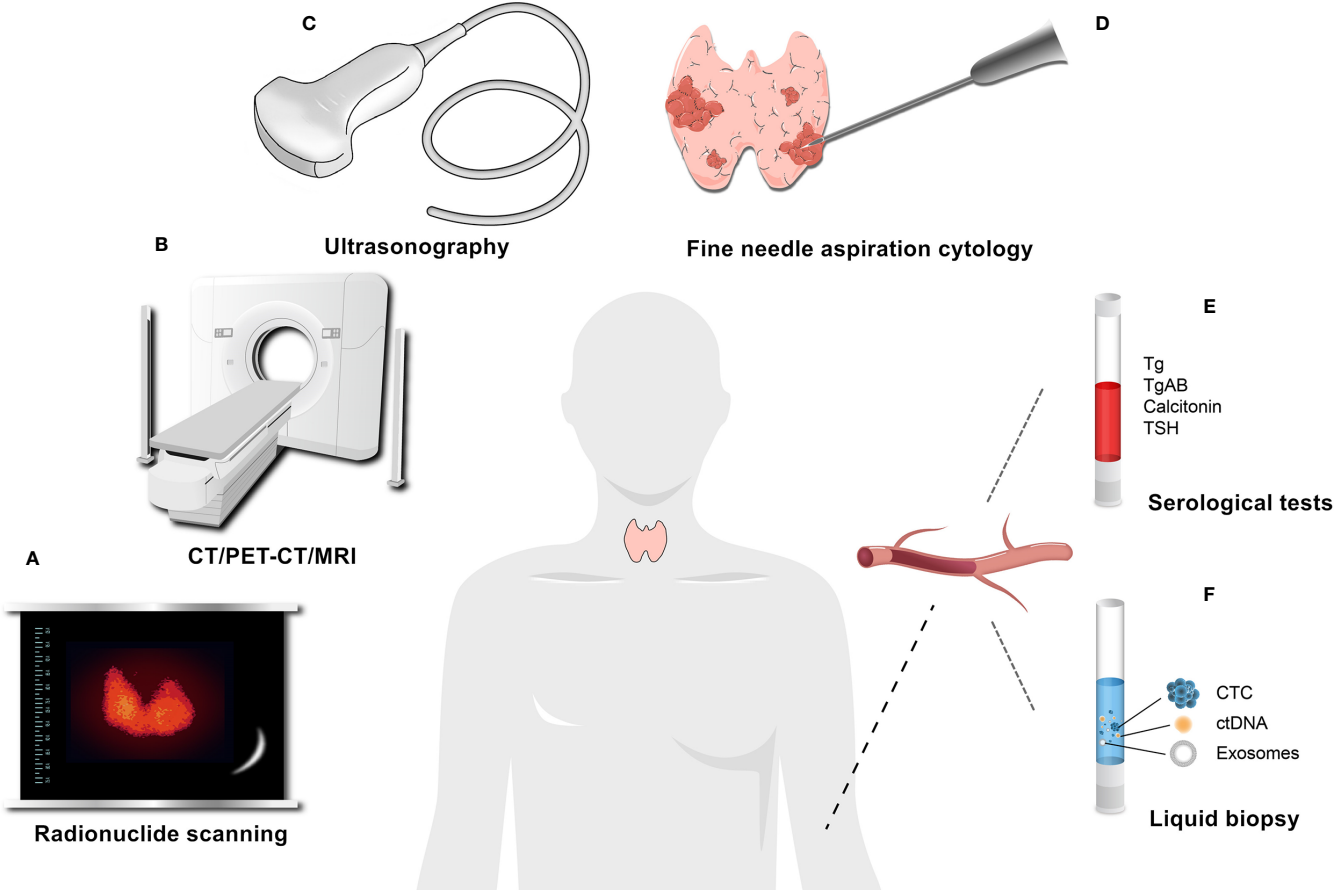
Common auxiliary examinations for thyroid cancer. **(A)** Radionuclide scanning. **(B)** CT/PET-CT/MRI. **(C)** Ultrasonography. **(D)** Fine needle aspiration cytology (FNAC). **(E)** Serological tests, such as Tg and Calcitonin, which are commonly used serum markers, can be used for the postoperative assessment and monitoring of DTC and the diagnosis and follow-up of MTC, respectively. **(F)** Liquid biopsy.

The diagnosis of TC is a challenging issue. Multiple methods, including radionuclide scanning, high-resolution ultrasonography, and fine needle aspiration cytology (FNAC), are used to diagnose TC (48) (**Figure 2**). FNAC is a reliable application but not perfect, and a small ratio of indeterminate samples results in unnecessary surgeries or missed diagnoses (49). In oncology, liquid biopsy is an alternative, complementary diagnostic and prognostic tool that can overcome some weaknesses of tissue biopsy. It lacks the pitfalls of invasive and nonrepeatable sampling and may have more advantages in safety, early diagnosis, and dynamic monitoring (50, 51). In liquid biopsies, compared with circulating tumor cells (CTCs), circulating tumor DNA (ctDNA), cell-free DNA (cfDNA), and cell-free RNA (cfRNA), exosomes are more likely to have unique advantages in patients sometimes (52, 53). For example, KRAS mutations can be detected in tumor exosomes at a higher rate than in cfDNA for patients with pancreatic ductal adenocarcinoma (54).

Recent studies have shown that exosomal microRNAs (miRNAs) are an appropriate and promising marker for the clinical diagnosis of tumors. The advantage of miRNAs in diagnosis is that they are highly stable, protected by a bilayer membrane, and contain key information related to the biological response of the tumor (55, 56). Lee and his collaborator found that the levels of miR-146b and miR-222 in TPC-1 exosomes were higher than those in NTHY cells, indicating that these two miRNAs may be biomarkers for PTC recurrence (57). Interestingly, another study detected plasma exosomes in PTC patients with or without lymph node metastasis (LNM), confirming that circulating exosomal miR-146b-5p and miR-222-3p have high diagnostic value for predicting LNM in PTC patients (58). Although most PTC patients have a good prognosis, LNM often occurs in the early stage, and predicting and diagnosing LNM in advance can prevent patients from experiencing unnecessary pain. The discrimination of follicular lesions is another difficulty in TC, and the current traditional diagnostic methods are often inadequate (52, 59). Samsonov et al. compared patients with benign thyroid nodules, and found that miR-31 in the serum exosomes of PTC patients was significantly upregulated. Moreover, miR-21 in the serum exosomes of FTC patients also showed the same changes. In addition, compared with FTC patients, PTC patients have reduced miR-21 levels in serum exosomes but significantly upregulated miR-181a-5p content. Therefore, these miRNAs in exosomes can be used as diagnostic markers for PTC and FTC (60). With the continuous improvement in high-throughput assays, more miRNAs were subsequently discovered. Wang ZY et al. conducted a plasma miRNA profile analysis on PTC patients and healthy subjects and then verified the experimental results. Among the candidate miRNAs, miR-346, miR-34a-5p, and miR-10a-5p were found to be upregulated in PTC plasma exosomes (61). Pan Q et al. isolated exosomes from the plasma of patients with PTC and nodular goiters through small RNA sequencing and a comprehensive analysis and identified a group of miRNAs in plasma exosomes as candidate biomarkers for the diagnosis of thyroid nodules; among these, miR-5189-3p had the best effect on diagnosing PTC (55). Dai D et al. discovered that miR-485-3p and miR-4433a-5p might serve as biomarkers for PTC diagnosis. Plasma exosomal miR-485-3p could enable discrimination between high-risk and low-risk PTCs (62). Li MH et al. screened a panel of miRNAs in plasma exosomes by analyzing exosomes from different patients and found that the combination of these miRNAs is more effective in identifying PTC and thyroid nodules than any single marker (63). These results suggest that the comprehensive detection of multiple exosome contents may be more advantageous.

Exosomes contain a large amount of ncRNAs that are involved in every stage of cancer progression. Circular RNAs (circRNAs) are a class of covalently closed endogenous biomolecules of ncRNAs. Special structural features make them highly stable (64). Under the protection of exosomes, circRNAs have been proposed as novel predictive biomarkers with clinical promise (65). Wu G et al. investigated the expression of circRNAs in PTC tissues and normal tissues by microarray analysis and demonstrated that circRASSF2 was upregulated in serum exosomes from PTC patients, suggesting the importance of circRASSF2 in tumor progression (66). Yang C et al. identified altered circRNA expression in serum exosomes from patients with PTC by high-throughput sequencing. circ_007293, circ_031752, and circ_020135 have been shown to be differentially expressed in the plasma of patients with PTC (67). In addition to serum exosomes, thyroglobulin in urine exosomes has also been found to be an emerging diagnostic marker for TC (68). These results suggest that the application of exosomes detected in TC could contribute to its diagnosis. However, due to the complex fluid composition of tumors, the dynamics of biomarkers, and the need for efficient enrichment and high sensitivity, the application of exosomes in cancer diagnosis remains challenging (26, 51). In recent years, the rapid development of nanotechnologies, including single‐exosome analysis used for the rapid and readable detection and molecular analysis of exosomes, may provide a great opportunity for expanding the use of exosomes (23, 51, 69). The combined application of multiple diagnostic methods, including exosomes, will accelerate the arrival of an era of more precise and refined thyroid diagnoses.

## TC-Derived Exosomes in Tumor Progression

**Figure 3:**
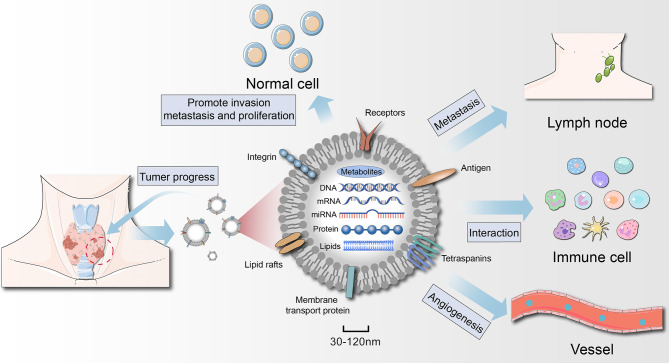
Role of thyroid cancer-derived exosomes in cancer progression. Exosomes act on tumor cells themselves, regulate angiogenesis, induce immunosuppression, and then remodel the tumor microenvironment to achieve tumor growth and metastasis. Exosomes secreted by thyroid cancer (thyroid CSCs) can also promote the oncogenic transformation of normal cells.

The development of tumors is a complex process, and malignant cells constantly interact with their surroundings (70). During this process, exosomes act as connectors and regulators to help the tumor grow. We have come to realize that the biological changes that occur in exosomes reflect their cell state (including oncogenic transformation) (11). A large body of evidence suggests that tumor cells often secrete more exosomes than do healthy cells, and the onset of cancer may cause changes in the molecular cargo of exosomes (71, 72). The number of exosomes is significantly higher in TC patients than in healthy subjects (53, 73), suggesting that exosomes are an important mediator in the progression of TC (**Figure 3**).

### The Role of Exosomes in TC Growth and Metastasis

An increasing number of studies have shown that exosomes can not only be used for clinical diagnosis and treatment, but also play an important role in the formation and metastasis of TC. Exosome-mediated intercellular communication plays a critical role in the development of cancer (74). Tumor cells actively produce, release, and utilize exosomes to promote tumor growth (72). As mentioned earlier, miR-146b and miR-222, which are relatively abundant in the exosomes of TPC-1 cells, caused a negative proliferative effect on both TPC-1 and NTHY cells (57). Jiang K et al. observed that exosomal miR-146b-5p and miR-222-3p were not only associated with LNM but also significantly enhanced the migration and invasion abilities of PTC cells (58). The exosome cargo continuously changes, and the quantity depends on the course of disease progression (75). For instance, high expression of exosomal miR-485-3p correlates with tumor size, extrathyroidal extension, the BRAF mutation, LNM and an advanced clinical stage (62). Ye W et al. revealed that miR-423-5p may be involved in the carcinogenic activities of TC. Silencing miR-423-5p in TPC-1 cells inhibited PTC cell migration and invasion owing to reduced miR-423-5p in exosomes, providing a new approach for the treatment of PTC (76). The substances in exosomes play a significant role in the formation and metastasis of TC. Wu G et al. indicated the oncogenic property of circFNDC3B in PTC cells; circFNDC3B was upregulated in serum exosomes from PTC patients compared with healthy patients and could modulate PTC progression by activating the miR-1178/TLR4 pathway (77). In addition, Wu G confirmed that circRASSF2 can exert oncogenic activity in the tumorigenesis of PTC by sponging miR-1178 to upregulate TLR4 expression (66). The metastasis and survival of tumor cells depend on the TME in addition to intrinsic changes in cancer cells. Exosomes released by cancer cells can alter the behavior of localized or recruited stromal cells *via* external interactions that shift the phenotype of these stromal cells into a metastatic one. This leads to the formation of premetastatic niches that promote tumor growth (71, 78). Cancer-related fibroblasts (CAFs) are the primary stromal cells in the TME and play an essential role in several processes in cancer biology (79). Exosomes released from CAFs contribute to tumor progression and metastasis by transferring substances to cancer cells, and exosomes secreted by cancer cells can promote CAF transformation (20, 80). The crosstalk between TC cells and CAFs has been continuously revealed, and the role of exosomes in their interactions is worthy of further investigation (81). The ongoing revelation of the underlying mechanisms and pathogenesis of TC progression, as well as the influence of exosomes on these mechanisms, may provide more strategies for eventually curing TC.

### Epithelial-Mesenchymal Transition (EMT) in TC

EMT is a cellular process during which epithelial cells acquire mesenchymal phenotypes and behaviors following the downregulation of proteins that indicate epithelial features (82). EMT arises early during tumor metastasis, playing a key role in mediating the development of an aggressive and invasive tumor phenotype. The main features of EMT include reduced adhesion and increased motility (83). EMT is triggered when a cell receives a signal from the microenvironment (82, 84). Studies have found a close link between EMT and cancer stem-like cells (CSCs), which are subsets of malignant tumor cells with the potential for self-renewal and differentiation and the ability to initiate tumors *in vivo* (85, 86). There are multiple ways by which EMT and CSCs interplay with each other (84, 87). Hardin et al. found that exosomes from thyroid CSCs transferred the long noncoding RNA (lncRNA) MALAT1, lncRNA ROR, and the EMT marker SLUG and induced EMT in normal thyroid cells (73). PTC-CSCs can also transmit the exosomal lncRNA DOCK9-AS2 to PTC, which can aggravate its malignant activities, including EMT, through the Wnt/β-catenin pathway (88). Critical components such as nucleotides, proteins, and organic intermediates in exosomes can serve as promising EMT regulators (89). Luo D et al. reported the differential expression of several proteins including the overexpression of proteins that are significantly related to cancer cell metastasis are associated with EMT (SRC, TLN1, ITGB2, and CAPNS1), in the exosomes of PTC patients with LNM (90). Some well-characterized miRNAs are important mediators involved in EMT-related signaling pathways induced by exosomes (91). Myriem et al. found that the overexpression of miR-145 in TPC-1 cells inhibited VEGF secretion and N-cadherin expression, which are closely related to the EMT process. Tumors actively excrete miR-145 into the blood *via* exosomes to reduce its expression in tumor tissue, thus inducing EMT and promoting the growth and metastasis of TC (92). Growing evidence has revealed that tumor cells are often in a different state along the EMT spectrum, as EMT is a continuous process with varying degrees of epithelial and mesenchymal characteristics and is often reversible (93, 94). We hypothesize that exosomes are involved in the dynamic changes between the epithelial and mesenchymal states. Future studies that show the inhibition of EMT-related changes and the reversal of EMT induction of tumors through exosomes will provide evidence of the use of exosomes to mitigate TC invasion and metastasis.

### Effect of Exosomes on TC Angiogenesis

The growth and metastasis of malignant tumors depend on the formation and dilation of blood vessels (95). Exosomes from various tumors, such as breast cancer (15), colorectal cancer (96), gastric cancer (97), and lung cancer (80), have been shown to play an important role in promoting angiogenesis. It should be noted that the exact mechanism of exosome-driven angiogenesis is not clear, and the angiogenic characteristics of exosome cargoes from different tumor cells vary widely (98). The thyroid is an important endocrine organ with abundant vascularity (99). In addition, there are more blood vessels in thyroid tumor tissue than in normal thyroid tissue (100). In cancerous tissues, angiogenesis is implicated not only in primary tumors but also in the formation and further outgrowth of metastases (101). Some conditions, such as hypoxia or acidosis, enhance the secretion of exosomes into bodily fluids (102). Tumors are often hypoxic, and this hypoxic state results in advanced but dysfunctional vascularization and can alter different aspects of tumor metabolism (103). Feng W and colleagues revealed that exosomal miR-21-5p secreted by hypoxic PTC cells enhanced the angiogenic activities of human umbilical vein endothelial cells (HUVECs) both *in vitro* and *in vivo*. Exosomal miR-21-5p is a potent proangiogenic factor that increases angiogenesis by inhibiting the expression of TGFBI and COL4A1. In addition, it was also found to be elevated in the serum of PTC patients (104). This enhanced angiogenesis and increased vascular permeability in tumors support tumor growth and metastasis by continuously providing sufficient oxygen and nutrients to meet the nutritional needs of subsequent rapid metastatic growth, thereby increasing the proliferation of cancer cells and the formation of a premetastatic niche (105, 106). Currently, some drugs, such as docosahexaenoic acid and curcumin, that can inhibit the angiogenic effects induced by exosomes have been reported (107, 108).

### Exosomes Mediate the Immune Regulation of TC

Cancer immune surveillance is considered a significant host protection process to maintain cell homeostasis and inhibit carcinogenesis (109). Cancer cells secrete exosomes to regulate the function and abundance of components in the TME, including immune effector cells and antigen-presenting cells, to escape their surveillance (110). In the microenvironment, exosomes also interact with tumor-associated macrophages (TAMs), which contribute to tumor growth, invasion, angiogenesis, and overall metastasis (105). Cancer-derived exosomes can act as cellular messengers to modulate macrophage polarization to the M2 phenotype, thus providing an immunosuppressive microenvironment for cancer cell survival and invasion (111). Macrophage infiltration is frequently detected in human thyroid tumors (112), and an increase in TAM density is positively correlated with the progression of TC, with TAMs accounting for more than 50% of nucleated cells in cases of ATC (113, 114). We suspect that TC can also affect TAMs through exosomes, but this hypothesis requires further study. Natural killer (NK) cells are recognized as particularly effective immune cells involved in immune surveillance because they are not restricted by the expression of MHC molecules (115). Exosomes from different tumor cells can not only be taken up by NK cells, but also inhibit NK cell cytotoxicity in several cancer types (116, 117). Cancer-derived exosomes induce NK cell dysfunction, inhibit antigen-presenting cells, block T cell activation, and enhance T cell apoptosis to block adaptive immune responses and suppress the antitumor immune response (118, 119). Additionally, the stimulation of NK cells, for example, with cytokines, can improve their tumor-killing effects. Zhu et al. demonstrated that exposure of NK cells to IL-15 results in the increased production of EVs and enhances the immunotherapeutic effects of EVs against several human cancers, including TC (120). Heat shock proteins (HSPs) can mediate immunomodulatory effects and the immune response (121). Caruso et al. found that the levels of Hsp27, Hsp60, and Hsp90 were increased in TPC tissue compared with normal peritumoral tissue and benign goiters. The levels of HSPs in the exosomes of patients with TPC before surgery were significantly higher than those in the exosomes from the same patients after surgery and from patients with benign goiters (122). TC is considered one of the most immunogenic cancers (123); thus, the rapid development of immunotherapy and exosomes as a delivery system brings new opportunities for the treatment of TC.

### The Potential of Exosomes in TC Treatment

The ability of exosomes to transport biomolecules to recipient cells has led to the increased exploration of exosomes as a tool in the field of cancer therapy (23). Elucidation of the role of exosomes in improving drug sensitivity, tumor radiosensitivity, and other responses to treatments will provide ideas for patients with advanced TC. Therapeutic applications of exosomes are promising because they have been shown to be well tolerated and have high bioavailability, low toxicity, and low immunogenicity (124). Notably, employing exosomes as a drug delivery system can easily avoid the first-pass metabolic effect and be effectively delivered to target sites. Compared to liposomes, exosomes can function better at a distance because of their superior systemic retention (125). Encapsulating anticancer drugs within exosomes has shown potential for targeted delivery to tumors in animal models. Gangadaran et al. isolated EVs containing Renilla luciferase in conditioned medium from cultured ATC cells (CAL62) to test the targeting ability of tumor-derived exosomes to their parental tumor in a mouse model. Exosomes injected into ATC model mice were directly internalized into CAL62 tumors and accumulated in the tumor region 30 min after injection (126). Previous studies have shown that cancer cells readily internalize EVs to a greater extent than normal cells. These results support the notion that cancer cell-derived exosomes harbor a specific tropism that can be exploited to deliver drugs into tumor cells. Despite the controversy, the ability of exosomes secreted by stem cells (such as MSCs) to inhibit tumorigenic properties has raised interest in research into their treatment of tumors (127). For instance, bone marrow MSCs can deliver exosomal miR-152 to repress the proliferation and migration of TC cells by targeting DPP4 (128). MSC-derived exosomes have many advantages over stem cells or synthetic nanoparticles, such as availability, ease of manipulation, and preferential access to tumors (24, 129). However, continuous efforts are still needed before exosomes can be applied clinically. Driven by chemical engineering and nanotechnology, *in silico* exosomes have been fully developed and are predicted to further promote the development of treatments.

The benefits of using exosomes to enhance antitumor immune responses in tumor microenvironment are also emerging. As mentioned above, the IL-15-mediated release of EVs by NK cells has an immunotherapeutic effect on TC (120). More experiments are underway; for instance, engineered macrophage-coated nanoparticles are a promising drug delivery strategy for treating triple-negative breast cancer (130). Exosomes derived from processed dendritic cells showed strong antitumor effects in inducing effective antigen-specific humoral and cellular immune responses (131). Clinical trials of exosomes as cancer vaccines are also underway with the help of immune cells (26). Another benefit of using exosomes therapeutically is that certain environmental niches may allow only exosomes to pass through (24). With the joint efforts of researchers from different disciplines, these nanoparticles will translate from the laboratory to the clinic faster and more efficiently (69).

## Conclusions and Perspectives

Exosomes promote the growth and development of TC *via* various strategies. The critical role of exosomes and their cargoes in TC has led to many insightful studies (**Table 1**). As discussed, tumor-derived exosomes can be used as a potentially valuable noninvasive diagnostic tool for TC in the field of liquid biopsy because they provide a broad diagnostic window for TC detection and monitoring, and future studies will aim to clinically validate promising treatments that may benefit more TC patients. In addition, work aimed at the clinical validation of promising treatments is ongoing. Although the field of exosome research has been deepened and enriched in recent years, the mechanistic understanding and clinical application of exosomes in TC are still lacking. Tumor immunity, drug resistance, radiotherapy sensitivity, and other aspects of TC are the primary difficulties associated with treating advanced TC, however, studies on the involvement of exosomes in these processes are rare. In addition to research on the cancer-promoting mechanism of tumor exosomes, for most TC patients with a good prognosis, the role of exosomes in tumor inhibition may provide ideas for the treatment of other tumors. Further research on exosomes will contribute to a more comprehensive and multidimensional understanding of TC. We also recognize that there is still a long way to go before exosomes are ready for clinical use, as the underlying mechanisms relating to exosome biogenesis, selective cargo loading, transport and function need to be clarified, and the best practices for isolating and identifying large numbers of high-quality vesicles for clinical use remain problematic (26). Considering the complexity of exosomes, more research is needed to deepen the understanding of exosomes and develop new diagnostic and therapeutic strategies for TC.

**Table 1:** Function of exosomal substances in thyroid cancer.

	Components	Histotype	Functions	Year	References
miRNA	miR-5189-3p	PTC	Biomarker	2020	(55)
	miR-16-2-3p, miR-34c-5p, miR-182-5p, miR-223-3p, miR-223-5p, miR-146b-5p	PTC	Biomarker	2020	(63)
	miR-485-3p, miR-4433a-5p	PTC	Biomarker, Progression	2020	(62)
	miR-146b-5p, miR-222-3p	PTC	Biomarker, Metastasis	2020	(58)
	miR-21-5p	PTC (hypoxic)	Promotes angiogenesis	2019	(104)
	miRNA423-5p	PTC	Biomarker, Migration, Invasion	2019	(76)
	miR-346, miR-34a-5p, miR-10a-5p	PTC	Biomarker	2019	(61)
	miR-21, miR-181a	PTC, FTC	Biomarker	2016	(60)
	miRNA-146b, miRNA-222	PTC	Biomarker, Proliferation	2015	(57)
	miR-145	PTC, FTC, ATC	Biomarker, Metastasis, Stemness	2014	(92)
lncRNA	lncRNA DOCK9-AS2	PTC CSCs	Proliferation, Migration, Stemness	2020	(88)
	lncRNA MALAT1, lncRNA ROR	Thyroid CSCs	Metastasis, Stemness	2018	(73)
circRNA	circFNDC3B	PTC	Biomarker, Metastasis, Proliferation	2020	(77)
	circRASSF2	PTC	Biomarker, Tumorigenesis	2020	(66)
	circ-007293, circ-031752, circ_020135	PTC	Biomarker	2019	(67)
proteins	Thyroglobulin	PTC, FTC	Biomarker	2020	(68)
	TSHR	PTC, FTC, ATC	Unknown	2019	(35)
	Hsp27, Hsp60, Hsp90	PTC	Biomarker	2019	(122)
	SRC, TLN1, ITGB2, CAPNS1	PTC	Invasion, Metastasis	2018	(90)

miRNA, microRNA; lncRNA, long noncoding RNA; circRNA, circular RNA; CSCs, cancer stem-like cells; TSHR, thyrotropin receptor; HSP, heat shock proteins.

## Author Contributions 

DY conceived the idea and provided guidance. KF wrote the manuscript, GD and DN completed the figures, and RM and LZ carefully reviewed the manuscript. HL and YT made critical revisions to the manuscript. All authors contributed to the article and approved the submitted version.

## Funding

This study was supported by the National Natural Science Foundation of China (81372863), the University Scientific and Technological Innovation Team Project of Henan Province (19IRTSTHN002), the Thousand Talents Science and Technology Innovation Leading Talents Subsidy Project of Central Plains (194200510011) and the Major Scientific Research Projects of Traditional Chinese Medicine in Henan Province (No.20-21ZYZD14).

## Conflict of Interest

The authors declare that the research was conducted in the absence of any commercial or financial relationships that could be construed as a potential conflict of interests.
